# Supplementary material for: Coupling between motor cortex and striatum increases during sleep over long-term skill learning
Source: eLife. 2021 Sep 10;10:e64303. doi: 10.7554/eLife.64303 (PMC8439654; doi:10.7554/eLife.64303)
Supplement: Transparent reporting form [file elife-64303-transrepform.docx]

***eLife’s* transparent reporting form**

We encourage authors to provide detailed information *within their submission* to facilitate the interpretation and replication of experiments. Authors can upload supporting documentation to indicate the use of appropriate reporting guidelines for health-related research (see [EQUATOR Network](http://www.equator-network.org/%20)), life science research (see the [BioSharing Information Resource](https://biosharing.org/)), or the [ARRIVE guidelines](http://www.plosbiology.org/article/info:doi/10.1371/journal.pbio.1000412) for reporting work involving animal research. Where applicable, authors should refer to any relevant reporting standards documents in this form.

If you have any questions, please consult our Journal Policies and/or contact us: [editorial@elifesciences.org](mailto:editorial@elifesciences.org).

**Sample-size estimation**

- You should state whether an appropriate sample size was computed when the study was being designed
- You should state the statistical method of sample size computation and any required assumptions
- If no explicit power analysis was used, you should describe how you decided what sample (replicate) size (number) to use

Please outline where this information can be found within the submission (e.g., sections or figure legends), or explain why this information doesn’t apply to your submission:

This study contains neural activity obtained from 6 rats, each recorded for multiple days (5-14 days) - this information is stated at the start of the **Results** section. This number of animals and days of recording was chosen to match the typical sample sizes reported in studies examining the evolution of neural activity during motor learning. We did not compute any power analysis to determine sample size.

**Replicates**

- You should report how often each experiment was performed
- You should include a definition of biological versus technical replication
- The data obtained should be provided and sufficient information should be provided to indicate the number of independent biological and/or technical replicates
- If you encountered any outliers, you should describe how these were handled
- Criteria for exclusion/inclusion of data should be clearly stated
- High-throughput sequence data should be uploaded before submission, with a private link for reviewers provided (these are available from both GEO and ArrayExpress)

Please outline where this information can be found within the submission (e.g., sections or figure legends), or explain why this information doesn’t apply to your submission:

We did not exclude any outliers or other data points. Number of animals, electrodes, and units are reported in the relevant **Results** section as well as in the relevant **Figures**. Comparison of reach-to-grasp learning curves were reported from 6 animals (Figure 1). Reports of automatic reaching behavior was reported from 2 animals (Supplemental Figure 1). Comparisons of behavioral changes with AP5/saline infusions were reported from 6 animals (Figure 1). Comparisons of local field potential dynamics were reported from 4 animals (animals implanted with microwires), across 36 days, and across 423 pairs of M1/DLS electrode pairs (Figure 2-3). Comparisons of spiking dynamics were reported from 6 animals, across 52 total days, from 1100 M1 units/579 DLS units and 3969 M1/DLS unit pairs (Figure 4-7, Supplemental Figure 2-8).

**Statistical reporting**

- Statistical analysis methods should be described and justified
- Raw data should be presented in figures whenever informative to do so (typically when N per group is less than 10)
- For each experiment, you should identify the statistical tests used, exact values of N, definitions of center, methods of multiple test correction, and dispersion and precision measures (e.g., mean, median, SD, SEM, confidence intervals; and, for the major substantive results, a measure of effect size (e.g., Pearson's r, Cohen's d)
- Report exact p-values wherever possible alongside the summary statistics and 95% confidence intervals. These should be reported for all key questions and not only when the p-value is less than 0.05.

Please outline where this information can be found within the submission (e.g., sections or figure legends), or explain why this information doesn’t apply to your submission:

All statistical methods are described in **Methods** section. Distributions or individual data points/learning curves are presented in the **Figures**, as well as mean +- SEM, when possible. Sometimes, details of distributions and individual data points are instead presented in **Supplemental Figures** (Supplemental Figures 2-4). Exact p-values, t-values, and DOF are always reported.

(For large datasets, or papers with a very large number of statistical tests, you may upload a single table file with tests, Ns, etc., with reference to sections in the manuscript.)

**Group allocation**

- Indicate how samples were allocated into experimental groups (in the case of clinical studies, please specify allocation to treatment method); if randomization was used, please also state if restricted randomization was applied
- Indicate if masking was used during group allocation, data collection and/or data analysis

Please outline where this information can be found within the submission (e.g., sections or figure legends), or explain why this information doesn’t apply to your submission:

Given the nature of the experiment, randomization of subjects to categories was not relevant and not present in the submission. For saline/AP5 infusions (Figure 1), experimenter was blinded to infused drug during data collection.

**Additional data files (“source data”)**

- We encourage you to upload relevant additional data files, such as numerical data that are represented as a graph in a figure, or as a summary table
- Where provided, these should be in the most useful format, and they can be uploaded as “Source data” files linked to a main figure or table
- Include model definition files including the full list of parameters used
- Include code used for data analysis (e.g., R, MatLab)
- Avoid stating that data files are “available upon request”

Please indicate the figures or tables for which source data files have been provided:

Where possible individual data points are presented in **Figures**. MATALB code used to generate figures, as well as processed data relevant to each figure will be made available where possible (embedded in article or uploaded to external repository).
